# Local distortion as a pitfall of iterative MLEM reconstruction in myocardial perfusion imaging – A phantom study

**DOI:** 10.1002/acm2.70140

**Published:** 2025-07-13

**Authors:** Mohsen Qutbi

**Affiliations:** ^1^ Department of Nuclear Medicine, School of Medicine Shahid Beheshti University of Medical Sciences Tehran Iran

**Keywords:** artifact, hot spot, image distortion, iterative reconstruction, maximum likelihood expectation maximization, pitfall

## Abstract

**Purpose:**

To provide insight into appearance of local distortion as a pitfall and to assess performance of MLEM reconstruction in presence of a high‐contrast object or hot spot and to quantify extent of involvement, specifically in myocardial perfusion imaging.

**Methods:**

A checkerboard image is reconstructed with MLEM with and without the presence of a high‐contrast region or hot spot to demonstrate pattern of distortion in near and distant locations around it. Then, a cardiac NCAT phantom is constructed without (“control”) and with a nearby hot spot or highly‐intense object (as lung lesion close to lateral wall of LV). An in‐house MLEM algorithm is implemented and utilized for reconstruction. Images are analyzed by creating error images and profile plotting.

**Findings:**

Pattern of distortion on a checkerboard image is like a two diagonal bands of the same width of the spot crossing perpendicularly. Higher relative intensity (5:1 vs. 2:1) results in more distortion both in extent and severity. Tomographic image of control NCAT phantom reveals an almost uniform intensity in lateral wall. However, as relative intensity of spot increases, distortion worsens. Circumferential curves of all walls are almost superimposed except for the wall close to object (or lateral wall of the LV).

**Conclusion:**

Presence of a hot spot or object creates distortion its own periphery during MLEM reconstruction. The object‐of‐interest is influenced if located in that region. Degree of distortion depends on relative intensity of hot object to background. Likewise, an artifactual defect is created in LV wall in myocardial perfusion imaging.

**Abbreviations**
MLEM: maximum likelihood expectation maximization
LV: left ventricle
S/M ratio: spot‐to‐myocardium ratio

## 1 INTRODUCTION

Algebraic reconstruction techniques have been developed to overcome the drawbacks of analytic ones (e.g., filtered back‐projection or FBP) tomographic reconstruction. These iterative techniques are well‐suited for the processing of digital images as opposed to the previous one in that discretization creates image artifacts. Over the past years, the use of FBP has been largely replaced by iterative techniques following the widespread availability of powerful computers and dedicated image‐processing software facilities. The maximum likelihood expectation maximization (MLEM) algorithm is one of the frequently used iterative technique for image reconstruction in nuclear medicine laboratories for single‐photon emission computed tomography (SPECT) and positron‐emission tomography (PET) images.^1^, ^2^, ^3^, ^4^, ^5^, ^6^ Briefly, the algorithm starts from an initial estimate, and then, by repeated cycles of forward and back‐projection processes, the estimate is updated and improved through comparing it with the measured data over several iterations. This algorithm has many advantages including faster convergence, better‐quality final image, and less computational load compared to other iterative algorithms. Despite all these favorable features, it has some shortcomings including the poor performance in reproducing finely‐detailed objects and edges as well as non‐uniform convergence and resolution recovery across the tomographic slice ^2^, ^3^, ^7^ Many of these drawbacks and pitfalls are well‐addressed in the literature. One of the potential pitfalls that is less investigated is the image degradation or distortion around a high‐contrast or “hot” object. In other words, the organ‐of‐interest, for example the left ventricle (LV) in nuclear cardiac images, is affected by the local distortion of the nearby hot spot. So, any structure in vicinity of that object is distorted in terms of shape and intensity. This pitfall is rarely reported in clinical images. However, this artifact has not been evaluated technically so far. Some believes that this might be related to the Gibbs effect as the culprit mechanism which is a well‐known phenomenon in signal processing. It occurs when a sharp‐edged signal (i.e., mathematically, jump discontinuity) is approximated with finite number of sinusoidal terms in Fourier series. Inexact reproduction of the signal creates many overshoots and undershoots in the periphery (rippling pattern).^8^, ^9^ This rippling pattern distort regions around a sharp‐edged object. In myocardial perfusion images, in the presence of a hot spot of any source –‐a lesion that is actively accumulating radiopharmaceuticals or retention of radioactivity in sub‐diaphragmatic structures like the bowels–‐ when residing closely to the LV of the heart, some distortion appears in the adjacent myocardial wall. This appearance can be a source of false‐positive perfusion defect and interferes with the clinical interpretation of images. It is commonly recognized that this artifact occurs during reconstruction using the FBP approach.^10^, ^11^ The problem was once considered to be caused by utilizing a ramp filter during FBP, however, later investigations identified radiation attenuation as the mechanism.^10^, ^11^ A widely held opinion among clinicians and practitioners in nuclear cardiology is employing MLEM technique for reconstruction of cardiac SPECT and PET images to resolve this artifact, at least partially, in order to avoid artifactual perfusion defects. This strategy is effective in most circumstances, but this artifact does occur even when reconstruction is performed by MLEM.^12^, ^13^, ^14^ Apart from the occurrence, the circumstances in which this artifact is more likely to appear (such as the relative intensity of hot spot against myocardial uptake) and the extent to which this artifact degrades the uptake pattern of myocardial walls have not been precisely specified. The present study aims to provide an insight into the appearance of this distortion as a potential pitfall and to assess the performance of MLEM iterative reconstruction in the presence of a high‐contrast object or hot spot that is occasionally encountered in myocardial perfusion images using simulation of computer‐generated images. In addition to visualization of the artifact (the extent and severity of distorted area around the hot spot), other characteristics including the extent of involvement of adjacent and other more distant myocardial walls is also quantified using realistic computerized phantoms.

## 2 MATERIALS AND METHODS

### 2.1 MLEM reconstruction

For reconstruction of images, projections are acquired by Radon transform with the following parameters: 360° arc and 1° angular sampling. During the forward projection process, the phantom is rotated and the summation of the values of each column of pixels is calculated. During rotation, bi‐cubic interpolation is employed. To achieve more accurate results that are generalizable to real tasks, a Poisson noise is added to all sinograms. Afterward, the noisy sinogram (or measured data) of each phantom undergoes reconstruction by the MLEM algorithm. For MLEM reconstruction, an in‐house implemented algorithm is utilized according to the following formula ^15^, ^16^, ^17^:

$${\hat{X}}^{\left(k+1\right)}={\hat{X}}^{\left(k\right)}\;\;\times \;R^{-1}\left\{\frac{S}{R\{{\hat{X}}^{\left(k\right)}\}}\;\right\}\;\times \;\frac{1}{R^{-1}\left\{\;1\;\right\}}$$

Where R and $R^{-1}$ are the operators of Radon transform (forward projection) and inverse Radon transform (or back‐projection) respectively. The ${\hat{X}}^{\left(k\right)}$ and ${\hat{X}}^{\left(k+1\right)}$ are the tomographic slices of iterations *k* and k+1. The latter is the newly updated estimate of ${\hat{X}}^{\left(k\right)}$. The sinogram or measured data, *S*, is divided by the sinogram of the estimate generated in each iteration and after normalization by term $R^{-1}\left\{\;1\;\right\}$, the correction factor is calculated and back‐projected (from projection space to image space) and then is applied to the current estimate or $X^{\left(k\right)}$.

### 2.2 Simulation of local distortion by hot spot

**FIGURE 1:**
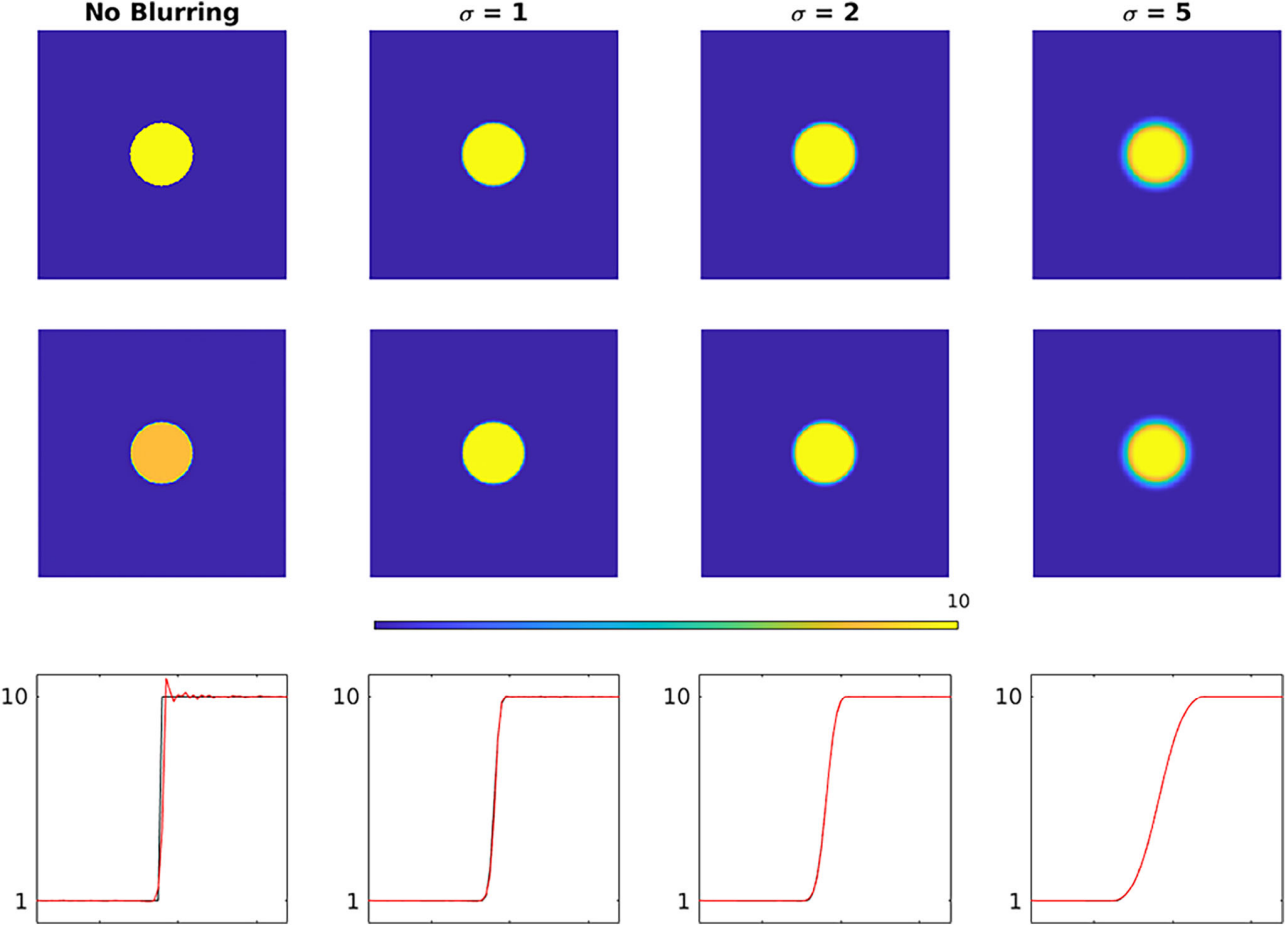
The images of objects without blurring and with increasing Gaussian blurring (specified by sigma in pixels) and corresponding reconstructed images and intensity profile plots. In the profile plots, the curves in black and red are related to the object and its reconstructed image respectively.

**FIGURE 2:**
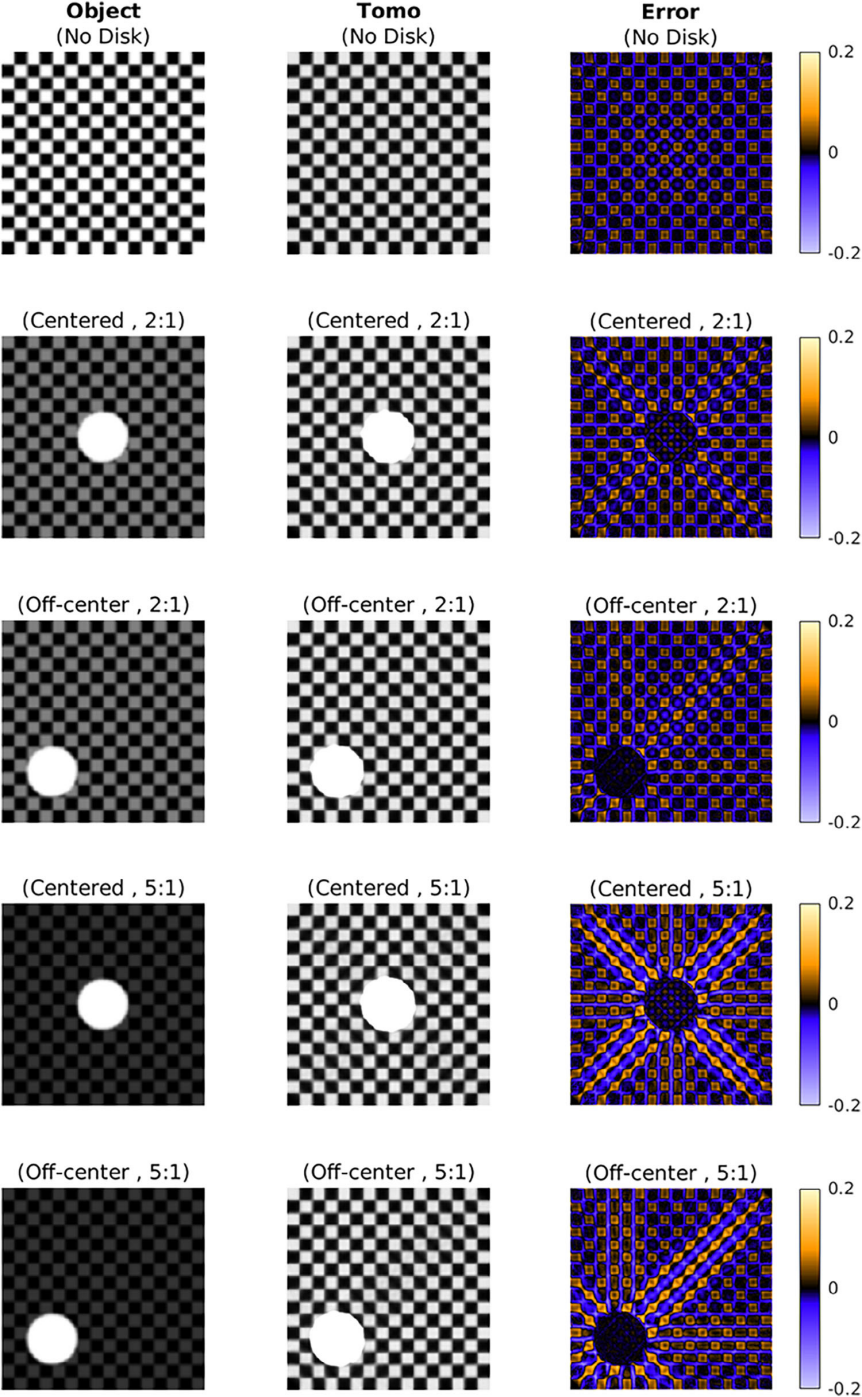
The checkerboard image as input to reconstruction procedure without any hot spot or high‐contrast disk (first row) and with a hot spot (high‐contrast disk) according to location (center and off‐center) and relative hot spot‐to‐background intensity (ratios of 2:1 and 5:1) in the second to fifth rows respectively as labeled.

To visualize and characterize the local distortion induced by hot spot, two initial simulations are conducted. First, a disk‐shaped object is used to visualize the Gibbs phenomenon in situations with various levels of edge blurring. For this task, the object with no blurring and different levels of Gaussian blurring (sigma of 1, 2, and 5 pixels) are reconstructed with MLEM method (as discussed above) and profile curves are plotted to demonstrate the rippling pattern (overshoots and undershoots) where edges are present (Figure 1). Then, in another task, an image with regular pattern of white and black rectangular regions (checkerboard pattern) is reconstructed with MLEM method with and without the presence of a high‐contrast region or hot spot in two different locations (center and periphery) to demonstrate the pattern of distortion in near and distant locations around the object (Figure 2). The procedure is conducted with two different values of intensity of hot spot compared to background (Ratio of 2:1 and 5:1) to assess the effect of relative intensity of high‐contrast object or hot spot on the degree of distortion. Then, the reconstructed images and the error images (difference between the original image and the corresponding reconstructed image) are visualized.

### 2.3 Simulation using cardiac phantom

**FIGURE 3:**
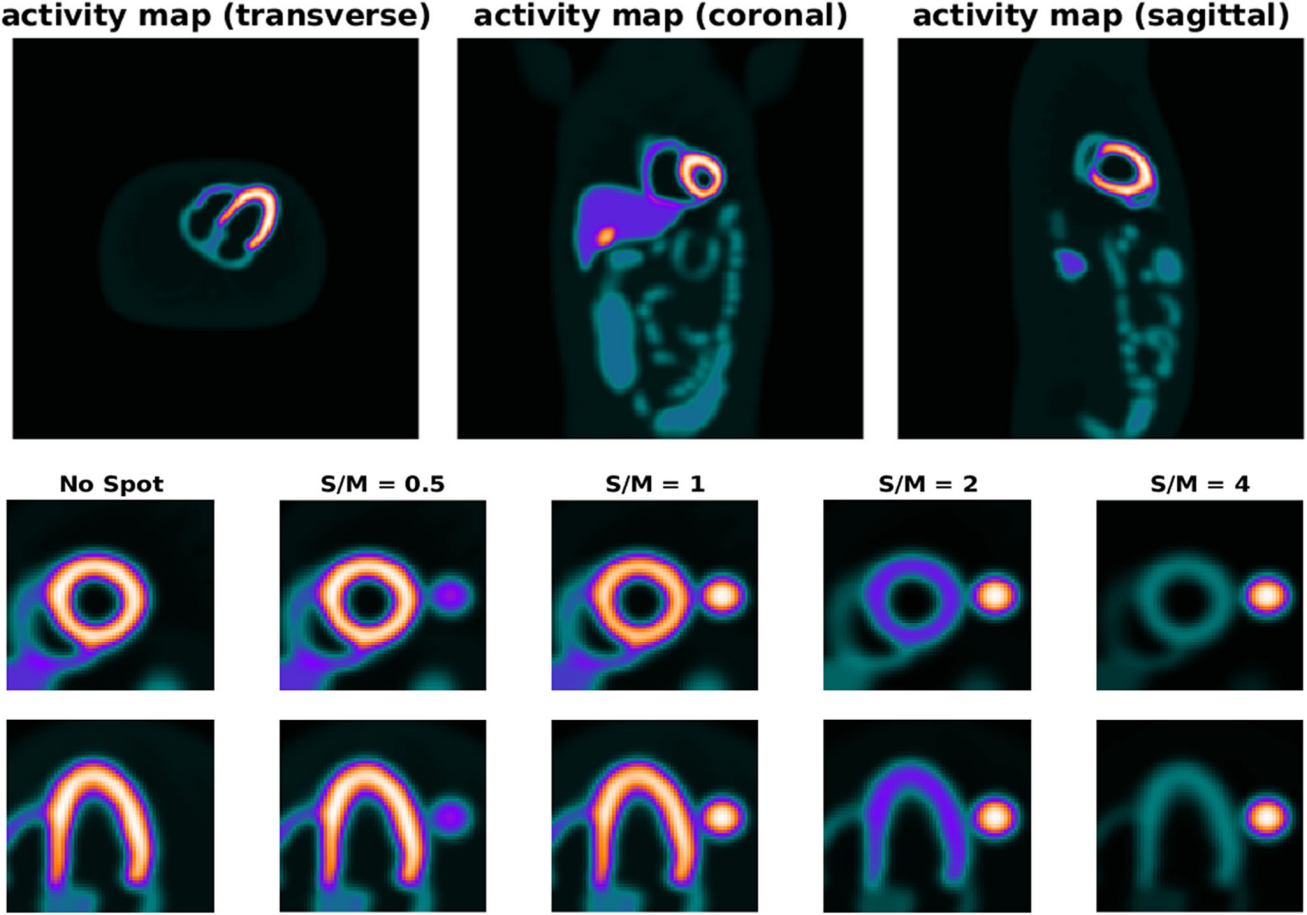
Upper panel shows the transverse, coronal, and sagittal sections of the NCAT phantom from left to right respectively. In the lower panel, the five phantoms with respect to the presence and relative intensity of the hot spot are demonstrated (short‐axis and horizontal long axis slices in upper and lower rows respectively, in lower panel). The leftmost is the control without any adjacent hot spot. Then, from left to right, are phantoms with hot spot with different S/M ratios (0.5–4). The images are scaled to the frame so that the relative intensity is observable and comparable.

An NCAT or 4D non‐uniform rational B‐splines (NURBS) ‐based Cardiac‐Torso phantom (version 2.0) ^18^ is used for construction of the cardiac phantoms. Using this phantom, values of activities are set based on the distribution of 99mTc‐Sestamibi radiotracer to simulate a myocardial perfusion scan. Emission or uptake map is generated in a matrix or array size of 256 by 256. A 2.34‐mm pixel size and slice thickness are determined. There are many other parameters for the construction of the phantom for a specified purpose. One can set the values as default or based on its specific application using the software. As such for the heart, there are various parameters including heart chamber size, length and radius, rotation in ZY, XZ, and YX planes, XYZ translation in the chest, cardiac motion, etc. The above mentioned quantities were used in selecting default values of the parameters of the phantom. The LV is configured with a length of 9.43 cm and a radius of 2.97 cm. The orientation of the heart is as follows: ZY rotation = −90.00°, XZ rotation = −20.00°, and YX rotation = −50.00°. The pixel value of the LV myocardium is arbitrarily set as 75 while that for the liver is set as 0.5 to avoid possible interference during image processing. This phantom is used as the “control” in which there is no nearby hot spot or highly intense object to the LV. Alongside, a 3.00 cm‐diameter spherical lung lesion is formed in the left lung, close to the lateral wall of the LV, with the goal of producing a hot spot. The 4 values of pixel intensity are chosen as 37, 75, 150, and 300 to simulate the spot‐to‐myocardium ratio (S/M Ratio) of 0.5 (half), 1 (equal), 2 (double), and 4 (quadruple). In addition to the first phantom (without a nearby hot spot), four other phantoms are generated using these lesions. The phantoms are presented in Figure 3. The transverse, coronal, and sagittal sections of the phantom are displayed in the upper panel and the short axis and horizontal long axis slices (which are reformatted after reorienting according to the long axis of the LV) are presented in the lower panel (upper row: short axis and lower row: horizontal long axis). The lung lesion as the hot spot adjacent to the LV is also seen with different values of S/M ratio. Then, the MLEM reconstruction is performed along the z‐axis of the phantom as the projections are acquired. In this way, the LV and the hot spot are present in the same section on which the reconstruction is conducted. Then, in order to propose a solution to avoid or resolve this artifact, the phantom is rotated +90° along the y‐axis. So the LV and the spot are located separately along the axis of reconstruction (i.e., not in the same sections). For the display of emission maps, a look‐up table or colormap of “cool” with 256 levels (8 bits) is used which is widely used for image display in nuclear cardiology. This colormap is extracted from FIJI ImageJ software and is imported into MATLAB.

### 2.4 Processing and analysis of cardiac images

Before processing and segmentation, all images are resized to acquire finer images (with an arbitrary scale of 6). For analysis of the tomographic images, the LV is segmented by the global thresholding technique (Otsu's method) in the control image and the boundary mask is applied to all other images. This approach is conducted for all transverse, short‐axis, and horizontal long‐axis slices as required. After segmentation, each slice is normalized according to the maximum pixel value in the region‐of‐interest of the LV (normalization to LV). For calculation of error, each image (with different S/M ratios) is subtracted from the control image (without hot spot) after normalization. For display of error a biphasic colormap (upper half: dark orange to light orange, lower half: dark blue to light blue and the middle zone as shades of black) is used to demonstrate both increase and decrease of pixel intensity all over the LV region‐of‐interest and the value of no changes is set to 0 (shown as black). The circumferential profile curves for horizontal long‐axis slices are plotted using sample points across the LV myocardial walls. In each horizontal long‐axis slice, the curves of control phantom and phantoms with different S/M ratios are plotted to demonstrate the changes in the intensity of myocardial walls.

### 2.5 Software and implementation

Image processing and analysis, including MLEM tomographic reconstruction, are implemented and conducted in MATLAB software package using image processing toolbox (The MathWorks Inc., version 2021b under Linux, Natick, Massachusetts, USA). The two colormaps utilized for visualization and display of images in this study are extracted from FIJI ImageJ software files of the colormaps (or lookup tables as.lut text file formats) and imported to MATLAB software.

## 3 RESULTS

**FIGURE 4:**
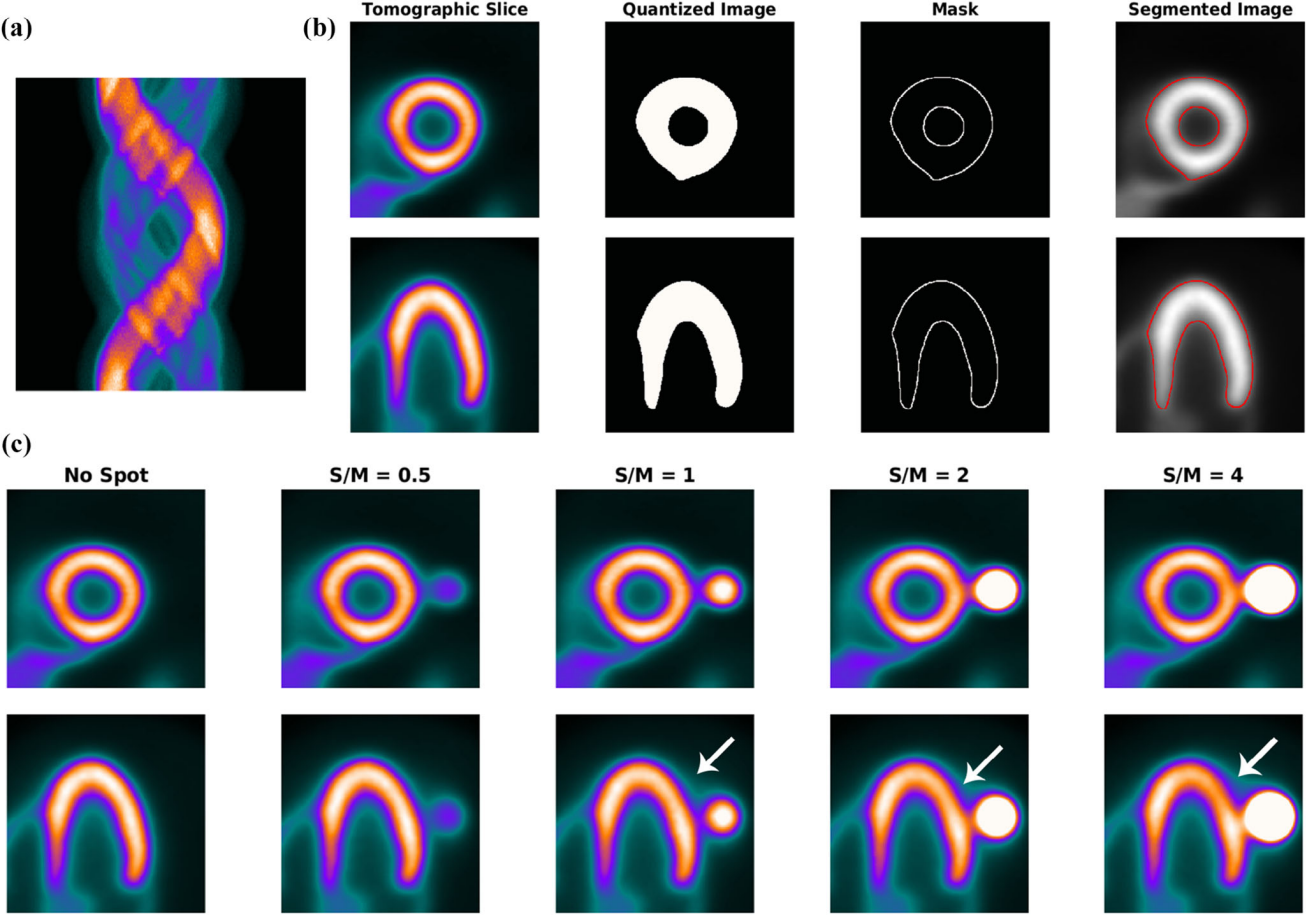
The sinogram or measured data of the original “control” phantom is presented in (a). In subfigure (b), the process of segmentation is demonstrated. The tomographic slices reconstructed by the MLEM technique, the quantized image, the mask, and the final image with contours or boundaries drawn are presented. The phantoms (control and four other ones with different S/M ratios) reconstructed by the MLEM technique and then reoriented along the long axis of the LV (short‐axis and horizontal long axis in upper and lower rows respectively) are displayed in C.

**FIGURE 5:**
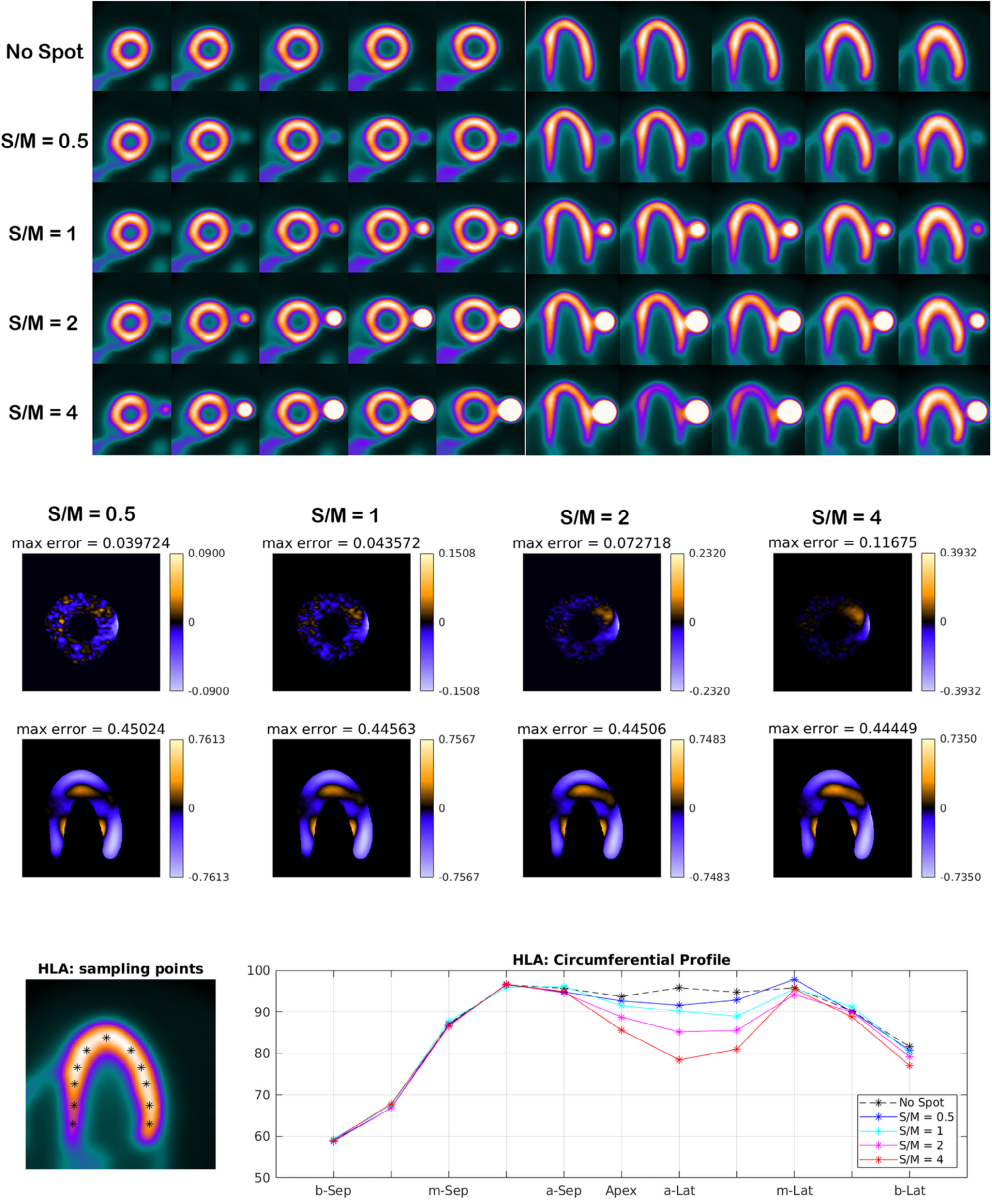
For each phantom with different S/M ratio, several consecutive slices (short‐axis and horizontal long‐axis) of the 3D phantom after reconstruction are presented (upper panel). Each row of images belongs to a phantom as labeled on the left side of panel. The error image for four phantoms with different S/M ratios (each subtracted from the image of the control phantom) is provided (middle panel) using a biphasic colormap. The circumferential profile curves of horizontal long‐axis (lower panel) for tomographic images of all five phantoms are presented. The curves are plotted based on the sample points (marked by asterisks in black) in the tomographic images (lower panel).

**FIGURE 6:**
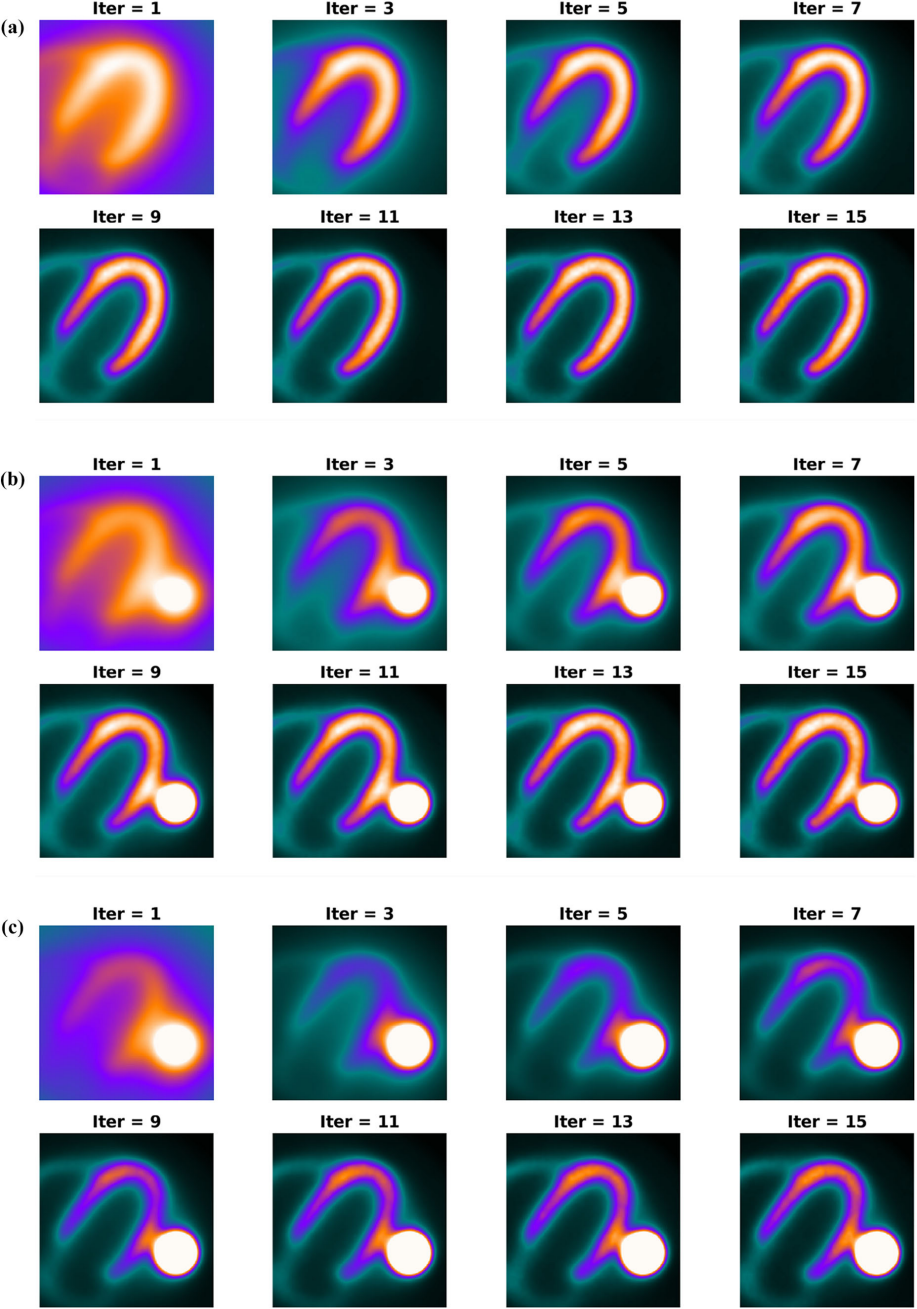
The reconstruction process of control phantom (a), phantom with S/M ratio = 2 (b), and phantom with S/M ratio = 4 (c) over consecutive iterations (from iteration 1 to iteration 15) are presented. For the control phantom, as visually detectable, the algorithm almost converges rapidly. B and C show the development and evolution of the defect in the lateral wall of the LV for the other two phantoms.

**FIGURE 7:**
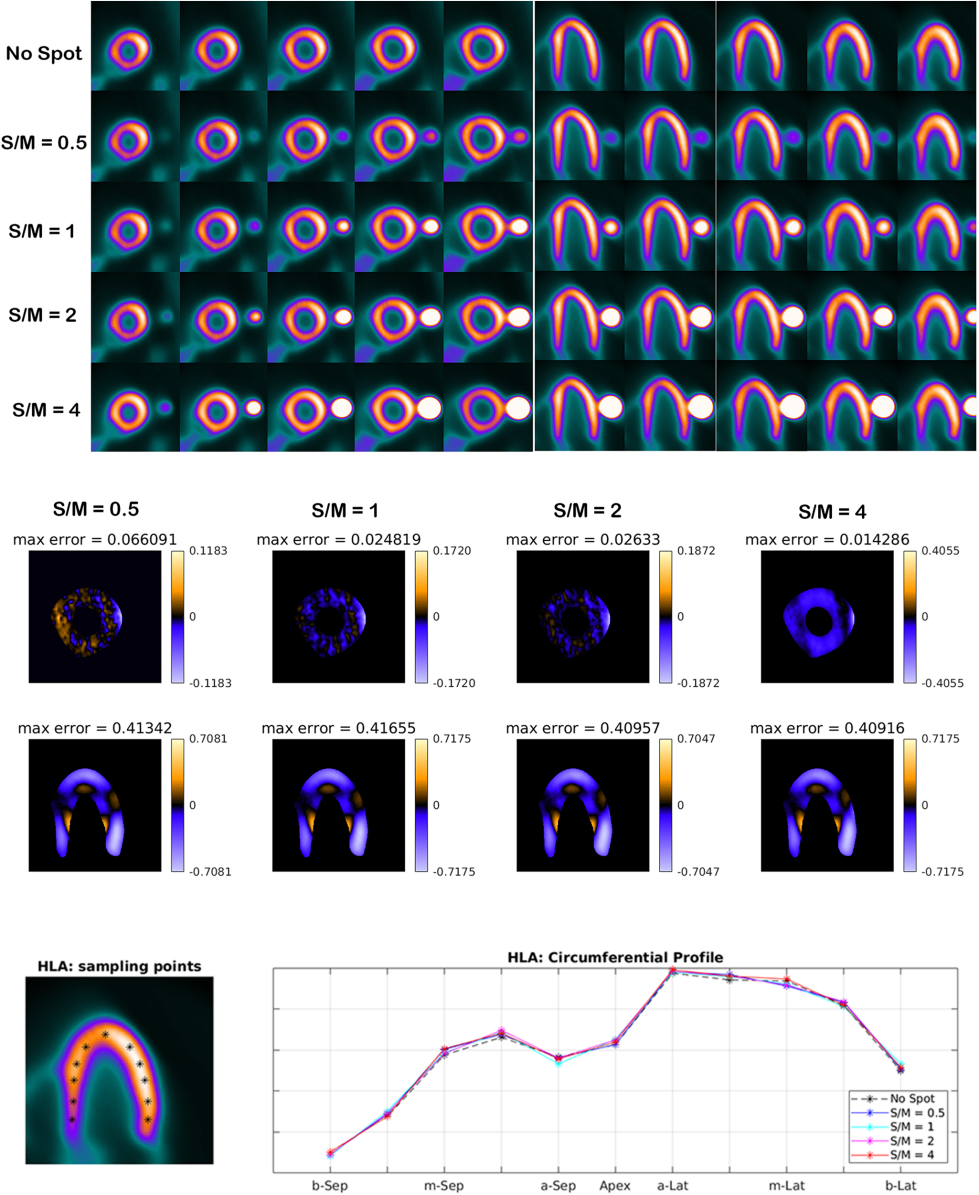
For each phantom with different S/M ratio, several consecutive slices (short‐axis and horizontal long‐axis) of the 3D phantom after reconstruction following the rotation of whole volume are presented (upper panel). Each row of images belongs to a phantom as labeled on the left side of panel. The error image for 4 phantoms with different S/M ratios (each subtracted from the image of the control phantom) is provided (middle panel). The circumferential profile curves of horizontal long‐axis (lower panel) for tomographic images of all five phantoms are presented. The curves are plotted based on the sample points (marked by asterisks in black) in the tomographic images (lower panel).

**FIGURE 8:**
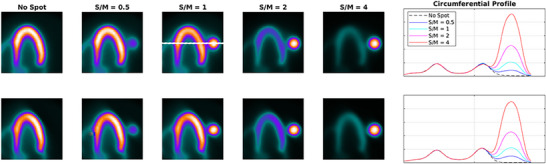
Profile curve across a horizontal line crossing the LV walls (septal and lateral) and also the (hot) spot for reconstructed images of all phantoms, shown on one of the images. The upper and lower rows of images and plots belong to the reconstruction procedure before and after volume rotation respectively.

As presented in Figure 1, in order to demonstrate the impact of Gibbs effect, a simple disk‐shaped object without blurring and with different levels of blurring specified by sigma as provided (the top row) is reconstructed by MLEM method (the middle row). To display the changes in the intensity in reconstructed images across the edge, intensity profile curve is plotted for each of them (the bottom row). As can be seen, the artifact induced by Gibbs effect is seen for the image without blurring where sharp edge is present. A distortion of pixel intensity is seen in the immediate region around the object. In other images (with blurring) the effect is no longer visible. The appearance of local distortion on a checkerboard image is provided in Figure 2. In each row, the image of object, reconstructed image and the error image are presented. In the first row, the object is reconstructed without hot spot region (used as control). In the second to fifth rows, the procedure is repeated with a hot spot (high‐contrast disk) according to location (center and off‐center) and relative hot spot‐to‐background intensity (ratios of 2:1 and 5:1). As can be seen, the distortion is visible in the white and black squares near and distant to the disk in two perpendicular directions. The width of distorted area is the same as the width of hot spot. Higher relative intensity (5:1 vs 2:1) results in more degradation and distortion both in extent and severity. In Figure 3, the phantom in orthogonal planes and the short‐axis and horizontal long‐axis of the LV in different situations of S/M ratio values are presented. By employing the forward projection process, the sinogram set for each of the phantoms is generated. As mentioned above, to make the procedure more realistic and analyze the true behavior of the MLEM algorithm, images are corrupted by Poisson noise. In Figure 4a, the noise‐corrupted sinogram of the control NCAT phantom is demonstrated. By using these sinograms, iterative reconstruction is performed for all phantoms. The procedure of segmentation for the control phantom as a case example is presented in Figure 4b. Then, these contours are used for reconstructed images of other phantoms (those with a neighboring hot object). This procedure is used for image scaling or normalization as presented in Figure 4c. The images are scaled to the maximum pixel value of the LV region‐of‐interest. As can be seen, the tomographic image of the control phantom reveals an almost uniform intensity in lateral wall. However, as the S/M ratio increases, the distortion worsens (indicated by arrows). In the S/M ratio of 4, a remarkable artifactual defect and non‐uniformity are seen along the lateral wall. The region of the LV wall closest to the object is artifactually intensified and conversely, neighboring regions in the same wall of the LV are suppressed. This pattern seems to be consistent for all phantoms but the severity becomes more striking. In this section, only a single slice (one short‐axis and one horizontal long‐axis) of each phantom after tomographic reconstruction is provided. In Figure 5 (upper panel), for each phantom with different S/M ratio, several consecutive slices (short‐axis and horizontal long‐axis) of the 3D phantom after reconstruction are presented. Each of the tomographic images of phantoms with a hot spot is subtracted from the control tomographic image in Figure 5 (middle panel), and thus error images are obtained. As described before, the highest distortion is in the lateral wall neighboring to the zone closest to the hot spot. The lateral wall in mid‐to‐apical regions is down‐scaled where the error is high and conversely, the zone in the mid‐to‐basal region, which is closer to the hot spot, is up‐scaled and when subtracted from the control image, a negative value of error is obtained. This means that this region shows higher intensity compared to that in the control image. Each error image is scaled to its own maximum. The pixels with zero changes are displayed in black. Those with positive and negative values of error (control image minus index image) are visualized in shades of orange and blue respectively. Apart from presenting error or discrepancy images, the analysis by profile plotting is also conducted (Figure 5, lower panel). Since the NCAT phantom is as much as a realistic model of the human body, the intensity of different walls is non‐uniform, therefore, the circumferential curves are not rather flat lines. The curves of all walls are almost superimposed except for the wall close to the object (or lateral wall of the LV), where the gap between the curve of the control phantom and the curves of phantoms with different S/M ratios widens. The graded increase of distortion is observed in the part of the curve that is related to lateral wall (more noticeably in mid‐to‐apical region of lateral wall). To visualize this artifact over consecutive iterations during MLEM reconstruction, tomographic slices obtained during odd iterations are displayed in Figure 6. This artifact is present from the first iteration, however, on later iterations, the influenced region becomes more sharply demarcated. The whole volume of each phantom is rotated in a way that the hot spot and the heart (or LV) are located along the z‐axis of the volume. Next, the reconstructed images/slices after volume rotation are provided in Figure 7 (**upper panel**). Interestingly, the defect in the myocardial wall is no longer present in each phantom with different S/M ratio. The lateral wall in all reconstructed images of different phantoms show uniform intensity or activity. Similar to that in Figure 5, the error images are computed (Figure 7, middle panel). By close inspection, the error images are almost identical which confirms the visual findings (in Figure 7 upper panel). Again, the circumferential curves of reconstructed images of phantoms are plotted (Figure 7 lower panel). All curves are almost overlapped and the widening gap between curves in lateral wall of the LV in no longer present. A profile curve across a horizontal line crossing the LV walls (septal and lateral) and also the (hot) spot for reconstructed images of all phantoms are plotted (Figure 8). The upper row images and plot belong to the reconstruction procedure before volume rotation and the lower row images and plot are for the experiment after volume rotation. As can be seen, in spite of the presence or absence of the defect, no photopenic halo (negative zone) is present around the hot spot (that might be related to Gibbs phenomenon). This finding is easily noticeable in the plots.

## 4 DISCUSSION

In nuclear medicine imaging, MLEM is a widely used iterative reconstruction method that has supplanted the FBP method. As previously described, in spite of all its favorable aspects, there are certain drawbacks.^2^, ^5^, ^7^ Among them, its performance in the presence of a hot spot has received less attention so far. In other words, the algorithm might not be able to effectively reproduce the changes in intensity in the spatial domain during reconstruction when there are zones with high pixel values. The intensity map becomes locally distorted or degraded as a result of this problem. It is worth noting that representing a signal with sharp edges (like a square wave) with a sum of the finite number of terms of Fourier series creates ringing or rippling pattern of many overshoots and undershoots with gradual decrements or increments, known as Gibbs phenomenon.^19^, ^20^ When no blurring is imposed, the effect is visible around the sharp edges of object. But when the level of blurring rises, this effect disappears. In addition, the maximum height of spikes (overshoots and undershoots) are limited, roughly 9% of the jump.^8^, ^9^ As seen in Figure 1, the width and severity of the effect is limited to a narrow rim on either side of the edge. Therefore, this effect is less likely to be a cause for noticeable local distortion around hot objects. The local distortion is elegantly visualized in Figure 2. When there is no hot spot, no distortion is seen in the regularly‐patterned image. However, presence of a hot spot causes some degree of distortion. The pattern is like a two diagonal bands of the same width of the spot crossing perpendicularly (in the error images). Therefore, if the object‐of‐interest is located in that specific regions will be influenced. Interestingly, the degree of distortion depends on the relative intensity of the hot object to the background. Similar observation is noticed in simulated images of cardiac phantom. The artifactual defect created in the adjacent region or wall of the LV in myocardial perfusion images discussed in this paper has significant clinical implications. In circumstances where such a hot spot is present, if retained activity in the bowels is the source of interference, one may attempt to use physiologic maneuvers to eliminate it.^21^, ^22^ Otherwise, for example in the presence of a lung lesion avidly accumulating radiotracer, no practical clinical solution exists. In most circumstances, using the FBP technique aggravates distortion. Therefore, it may be so challenging for the interpreting physician. For these reasons, we made an effort to simulate this situation and assess the performance of the MLEM reconstruction method. In this study, a cardiac phantom is created utilizing NCAT phantom which is a realistic 4D model of human anatomy and physiology. This serves as the control, and a lung lesion is subsequently made in the left lung of the phantom just close to the lateral wall of the LV. In order to simulate the impact of different levels of S/M ratio, different values of intensity or activity are defined in each experiment of this study while the size of the lesion and the distance between the lesion and the LV (as the organ‐of‐interest) are assumed fixed (Figure 3). This task is done to evaluate the effect of the relative intensity of LV myocardium and the hot spot. Do the extent and severity of the defect in the myocardial wall differ? To evaluate the images visually and to plot the curves, reorientation of the volume was required to obtain appropriate slices of the cardiac section (short axis and long axis). So, reorientation is carried out in accordance with the orientation and rotation of the heart in the thorax in various pairs of xyz planes. Before the volume was reoriented, the reconstruction process was completed. In fact, each transverse slice along the z‐axis is reconstructed separately. As observed in the reconstructed slices, the original intact distribution of activity or pixel intensity is seen in the control reconstructed slice. However, there is a considerable distortion in the original distribution of reconstructed slices of the other four phantoms with a hot spot, particularly when the S/M ratio is 1 or greater. The larger the S/M ratio, the higher the severity and the greater the extent of the defect as indicated by arrows in Figure 4. This pattern was also depicted in error images. The circumferential curves also demonstrated this pattern across different walls of the LV (Figure 5, lower panel). As illustrated in Figure 6, the process of formation of the defect or distortion is visualized in successive iterations during MLEM reconstruction. It is evident that this distortion appears to a remarkably higher extent from the first iteration. However, as the iterations go on, the degree of distortion converges and becomes more defined and demarcated. After rotating the whole volume of phantom in order to placing the LV (organ‐of‐interest) and the hot spot along the axis of reconstruction, the artifact on the lateral wall in no longer present after MLEM reconstruction (see Figure 6). Because the LV and the spot are placed in different section during forward projection and reconstruction procedures, and thus, the impact of the hot spot will not present on the organ‐of interest. This could be a potential solution for resolving or avoiding the artifact. However, this not practical in most cases since it requires acquiring images (projections) along the axis connecting these two objects. However, it works for nonhuman phantoms and small animals by meticulous placement of the animal on the scanning table or bed (analogous to the rotation of the phantom in our computer simulation). Although the best technique is removing the hot spot, this is a real challenging problem in clinical setting. In this study, the measured data or sinograms are corrupted with noise in order to incorporate the potential undesirable effect of noise. However, other physical factor like radiation attenuation is not modeled. These findings indicate that the distortion results from the procedure of image processing and reconstruction itself. In other words, mechanisms like radiation attenuation that contribute to the production of sinograms are irrelevant to this artifact. Another limitation of this study is that, we implemented the basic/standard version of MLEM algorithm. However, other modified versions provided by the vendors in commercially available software packages for cardiac images to improve resolution, signal‐to‐noise ratio and etc. may have beneficial or even adverse effect regarding this artifact and thus needs further assessment.

## 5 CONCLUSION

The local distortion around hot spots or high‐contrast objects are unlikely to be caused by Gibbs effect. The pattern of distortion is like a two diagonal bands of the same width of the spot crossing perpendicularly. Therefore, if the object‐of‐interest is located in that specific regions will be influenced. The degree of distortion depends on the relative intensity of the hot object to the background. Similarly, the presence of a hot spot or object creates an artifactual defect in the adjacent region of organ‐of‐interest (LV wall close to the hot lesion) during reconstruction by the MLEM algorithm. The severity and extent of the defect are related to the relative intensity of the hot spot and the organ‐of‐interest (LV myocardial wall).

## AUTHOR CONTRIBUTIONS

All parts of the project, from conception, design, data analysis, interpretation, and drafting is carried out by Mohsen Qutbi.

## CONFLICT OF INTEREST STATEMENT

The authors have no conflict of interests to disclose.

## Data Availability

Authors will share data upon request to the corresponding author.
